# Personalized Venetoclax Dose Adjustment in Unfit Acute Myeloid Leukemia Patients: A Real-Life Case Series Study

**DOI:** 10.3390/jpm16040200

**Published:** 2026-04-02

**Authors:** Serena Luponio, Bianca Serio, Idalucia Ferrara, Andrea Gigantiello, Anna Maria Della Corte, Denise Morini, Italia Conversano, Francesco Verdesca, Francesca Velino, Anna Maria Sessa, Simona Caruso, Rossella Marcucci, Martina De Leucio, Valentina Giudice, Maddalena Langella, Carmine Selleri

**Affiliations:** 1 Hematology and Transplant Center, University Hospital “San Giovanni di Dio e Ruggi d’Aragona”, 84131 Salerno, Italycselleri@unisa.it (C.S.); 2 Department of Medicine and Surgery “Scuola Medica Salernitana”, University of Salerno, 84081 Baronissi, Italy

**Keywords:** acute myeloid leukemia, WT1, minimal residual disease, personalized medicine

## Abstract

**Background/Objectives**: Minimal residual disease (MRD) negativity is associated with improved outcomes in acute myeloid leukemia (AML) patients. In this retrospective observational real-life case series study, we investigated the efficacy and safety of venetoclax dose adjustment in unfit AML patients and the role of WT1 expression levels as a surrogate marker of MRD monitoring. **Methods**: A total of 24 consecutive unfit AML patients treated with azacytidine and venetoclax were enrolled in this study, and MRD monitoring was performed by flow cytometry as per international guidelines and by WT1 expression levels assessed by RT-qPCR. Dose adjustment of venetoclax was decided based on MRD status and the onset of grade > 2 neutropenia. **Results**: The overall response rate was 87.5%, and 16 patients achieved a response already at the first re-evaluation (66.7%). No statistically significant differences were observed between patients who received the standard dose and those with venetoclax dose adjustment in terms of overall survival (19.6 months vs. 30.1 months, respectively; *p* = 0.9428) and progression-free survival (not reached vs. 22.1 months, respectively; *p* = 0.3865), although a numerical trend toward lower relapse rates was observed in subjects with late (33.3%) or early and late dose reduction (37.5%) compared to those who had dose adjustment only at the first re-evaluation (75%) (*p* = 0.3014). The toxicity rate was 33.3% in patients who had early and late dose adjustments, which was lower than that observed with early adjustment (58.3%) and than that reported in the VIALE-A study (84%). **Conclusions**: Reduced-dose venetoclax regimens (from 28 to 21 days per cycle) in unfit AML patients do not affect response rates or survival and are associated with comparable rates of neutropenia and infectious events, supporting flexible dosing strategies based on patient response and side effects. In addition, WT1 expression could serve as a reliable marker for MRD monitoring.

## 1. Introduction

Acute myeloid leukemia (AML), a clonal hematological malignancy, is characterized by uncontrolled proliferation of myeloid progenitor cells and a block in differentiation, resulting in the accumulation of leukemic cells in the bone marrow (BM) and peripheral blood (PB), ultimately leading to progressive pancytopenia [1]. AML is the most common hematological malignancy in adults in Western countries, accounting for ~3% of all cancers and 25% of all leukemias, with a higher incidence in males and a mean age of 69 years, with incidence increasing with age [2,3]. More than half of AML patients are aged >65 years, and approximately one third are >75 years old, and these subjects usually experience poor prognosis, as conventional induction therapies result in complete remission (CR) rates of 45–55%, and less than 10% of patients treated with aggressive therapy are alive at 5 years [4,5,6]. These unfavorable outcomes are due to several factors, including concomitant comorbidities, which worsen the toxicity of chemotherapy and transplantation or contraindicate their use, and the higher incidence of negative biological characteristics such as unfavorable cytogenetics or the secondary nature of AML following previous hematopoietic disorders, particularly myelodysplasias [6,7,8].

Decitabine and azacitidine, two hypomethylating agents (HMAs), are commonly used as induction therapy in the elderly; however, their use as a single agent is associated with a low remission rate (~30%) and a median survival of less than one year [9]. The addition of venetoclax, the first-in-class BCL2 inhibitor, to HMAs has revolutionized the treatment of AML patients unfit for induction chemotherapy, allowing an increase in survival and becoming the new standard of care in this subset of subjects [10]. The VIALE-A study has demonstrated that the addition of venetoclax to HMAs can dramatically improve clinical responses (66.4% vs. 28.3%) and median overall survival (14.7 vs. 9.6 months) [11]. However, response duration greatly varies, being only a few months in high-risk AML with *TP53* mutation and in AML with monocytic differentiation. Indeed, although the composite complete response rate greatly improves after venetoclax addition (55.3% vs. 0%) in these high-risk subjects, no survival benefit has been observed in the VIALE-A study [12].

As per the approved dosing schedule in AML, venetoclax is initiated at 100 mg on day 1, 200 mg on day 2, and 400 mg from day 3 and beyond for 28 days in each therapeutic cycle, in association with azacitidine at 75 mg/m^2^ of body surface area (BSA) either intravenously or subcutaneously on days 1–7 of each 28-day cycle beginning on cycle 1 day 1, or with decitabine at 20 mg/m^2^ of BSA intravenously on days 1–5 of each 28-day cycle beginning on cycle 1 day 1 [13]. Venetoclax dosing may be interrupted as needed for management of hematologic toxicities and blood count recovery and should be continued until disease progression or unacceptable toxicity occurs [14]. Indeed, in clinical practice, there is accumulating evidence that a fixed venetoclax dose and duration may be excessive for certain patients, leading to prolonged myelosuppression and increased risk of infection, ultimately leading to febrile neutropenia and death [15,16,17]. Retrospective studies have suggested that shorter venetoclax courses (7 or 14 days) may be equally effective, especially in frail patients or those who achieve a rapid response [18,19]; however, the lack of prospective trials comparing different dosages and durations does not allow the definition of the optimal approach in unfit AML patients. Guidelines recommend starting with a full course and modulating the duration of venetoclax based on early assessed response to therapy [20]. In subsequent cycles, the duration of treatment may be further shortened in the presence of prolonged myelosuppression and optimal disease control [10,21,22].

Minimal residual disease (MRD) assessment is a reliable tool for improving the outcome of AML patients, as MRD negativity is associated with improved survival [23]. The 2022 European LeukemiaNet (ELN) guidelines regulate its use in patients treated with intensive regimens, while no definitive recommendations are present for patients undergoing non-intensive venetoclax-based therapy [23,24]. Data on MRD in these patients are still limited; however, its monitoring could play a complementary and prognostically relevant role, even if MRD negativity may occur later on during intensive regimens [25,26,27]. Sensitive and specific MRD monitoring in elderly AML patients could permit dose adjustments to minimize drug-induced toxicities while not affecting therapeutic efficacy, thus favoring a personalized approach in these frail subjects [28,29].

For these reasons, in this retrospective observational real-life case series study, we aimed to (i) provide additional evidence on the effective and safe use of reduced venetoclax duration from 28 to 21 days per cycle in unfit/frail AML patients not eligible for intensive chemotherapy; and (ii) investigate the potential role of circulating WT1 expression levels as a surrogate marker for MRD monitoring when other disease-specific molecular alterations are present. These MRD-based venetoclax dose adjustments support a personalized therapeutic approach in unfit/frail patients in a real-life setting, to maximize clinical efficacy while minimizing treatment-related toxicities.

## 2. Materials and Methods

### 2.1. Patients

A total of 24 consecutive patients followed at the Hematology and Transplant Center of the University Hospital “San Giovanni di Dio e Ruggi d’Aragona” in Salerno, Italy, from March 2022 to October 2025 were enrolled in this retrospective observational real-life case series study. Patients received a diagnosis of AML according to the 2022 World Health Organization criteria [30], and risk stratification was performed based on the 2022 ELN guidelines [21]. Clinical characteristics are summarized in Table 1. Inclusion criteria were age ≥ 18 years, a confirmed diagnosis of AML, and provision of written informed consent. This study was conducted in accordance with the Declaration of Helsinki, the International Conference on Harmonization Good Clinical Practice guidelines [31], and protocols approved by our Ethics Committee “Campania Sud,” Brusciano, Naples, Italy (prot./SCCE no. 24988). Written informed consent was obtained from all participants. All patients received azacytidine plus venetoclax, according to the technical data sheet indications, and venetoclax dosage was reduced when administered in combination with posaconazole or according to the severity of hematological toxicity [13]. Venetoclax dosage was reduced in patients after MRD negativity was achieved or in the case of severe grade III-IV neutropenia. MRD monitoring was carried out by flow cytometry immunophenotyping and by circulating WT1 expression levels by RT-qPCR assay after every cycle of therapy and at relapses. MRD negativity was defined according to the 2022 ELN guidelines [32].

Toxicities were assessed according to the Common Terminology Criteria for Adverse Events (CTCAE) v.5.0. Responsiveness to treatment was estimated according to the 2022 ELN criteria: complete response (CR) with blast percentage < 5%; complete response with incomplete hematological recovery of white blood cells and platelets (CRi); partial response (PR) with blast persistence between 5 and 20%; stable disease (SD) with an unchanged percentage of blasts or increased <25% from baseline; or progression (PD) with blast increase >25% from baseline [21].

### 2.2. WT1 Quantitative Assessment

WT1 expression levels were quantified by real-time polymerase chain reaction (RT-PCR) at diagnosis, after each azacytidine-venetoclax cycle, and during follow-up. Mononuclear cells were freshly isolated from PB or BM samples by density gradient centrifugation using Lymphoprep (Axis-Shield Density Gradient Media, Oslo, Norway) and subsequently subjected to RNA extraction using the QIAamp RNA Blood Mini Kit (Qiagen, Hilden, Germany), following the manufacturer’s instructions. After RNA quantification, at least 1 µg of RNA was used for cDNA reverse transcription (Ipsogen RT Kit; Qiagen), and WT1-mRNA quantitative assessment was performed using an ELN-certified Ipsogen WT1 ProfilQuant Kit (Qiagen) following the manufacturer’s instructions. WT1 levels were reported as WT1 copy number/10^4^ ABL copies (normalized WT1 expression), and normal expression levels were considered <50 or <250 copies in PB or BM specimens, respectively, as previously reported [32,33,34,35,36].

### 2.3. Flow Cytometry

For immunophenotyping, 50 µL of fresh heparinized whole PB or BM was stained with antibodies according to the manufacturers’ instructions, and as previously described [33]. The following antibodies were used: CD3, CD5, CD7, CD8, CD11b, CD13, CD14, CD16, CD19, CD33, CD34, CD45, CD56, HLA-DR, CD117, CD71, SmIg-kappa, and SmIg-lambda. CD45dim cells were further characterized as previously described [32]. Briefly, after 20 min incubation at room temperature, red cell lysis was performed with IO Test Lysing Solution (Beckman Coulter, Brea, CA, USA), cells were washed twice with phosphate-buffered saline (PBS) (IsoFlow Sheath Fluid; Beckman Coulter), resuspended in 500 µL of PBS, and samples were acquired on a Navios/EX or DxFlex cytometer (Beckman Coulter). Daily instrument quality control was carried out using Flow-Check Pro Fluorospheres (Beckman Coulter), and external quality control by UK NEQAS for Leucocyte Immunophenotyping. Samples were run using the same PMT voltages, and at least 200,000 events were recorded. Post-acquisition analysis was carried out using Kaluza Analysis Flow Cytometry Software v2.1.1 (Beckman Coulter) and as previously reported [32].

### 2.4. Statistical Analysis

Data were collected in a computerized database and analyzed using GraphPad Prism (v 10.6.1; GraphPad Software, San Diego, CA, USA). Survival analysis was carried out using the log-rank Kaplan–Meier test. Differences between groups were studied using the Chi-square, Fisher’s, Wilcoxon signed-rank or unpaired two-tailed *t*-test tests. Missing data were handled using pairwise deletion. A *p*-value < 0.05 was considered statistically significant.

## 3. Results

### 3.1. Clinical Characteristics

The majority of patients in our cohort received a diagnosis of myelodysplastic syndrome (MDS)-related AML (*N* = 16, 66.7%) or de novo AML (*N* = 7, 29.2%), and most presented with adverse-risk disease (*N* = 16, 62.5%), according to the 2022 ELN risk stratification criteria. Four patients (16.7%) were previously treated with low-dose cytarabine + gilteritinib (*N* = 1), cytarabine + daunorubicin (*N* = 1), or liposomal cytarabine + doxorubicin (*N* = 2). At diagnosis, median WT1 copy number in the PB was 1388 copies/10^4^ ABL copies (range, 38–41,660 copies/10^4^ ABL copies) and in the BM was 2815 copies/10^4^ ABL copies (range, 28–6501 copies/10^4^ ABL copies). At the time of data cut, relapse rate was 37.5% (*N* = 9), and mortality rate was 66.7% (*N* = 16), mainly due to disease progression (*N* = 11, 45.8%), cardiovascular complications (*N* = 2; 8.3%), sepsis or acute respiratory distress syndrome (*N* = 2; 8.3%), or a secondary malignancy (*N* = 1; 4.2%).

### 3.2. Clinical Outcomes in Patients with Reduced Venetoclax Dose

**Figure 1:**
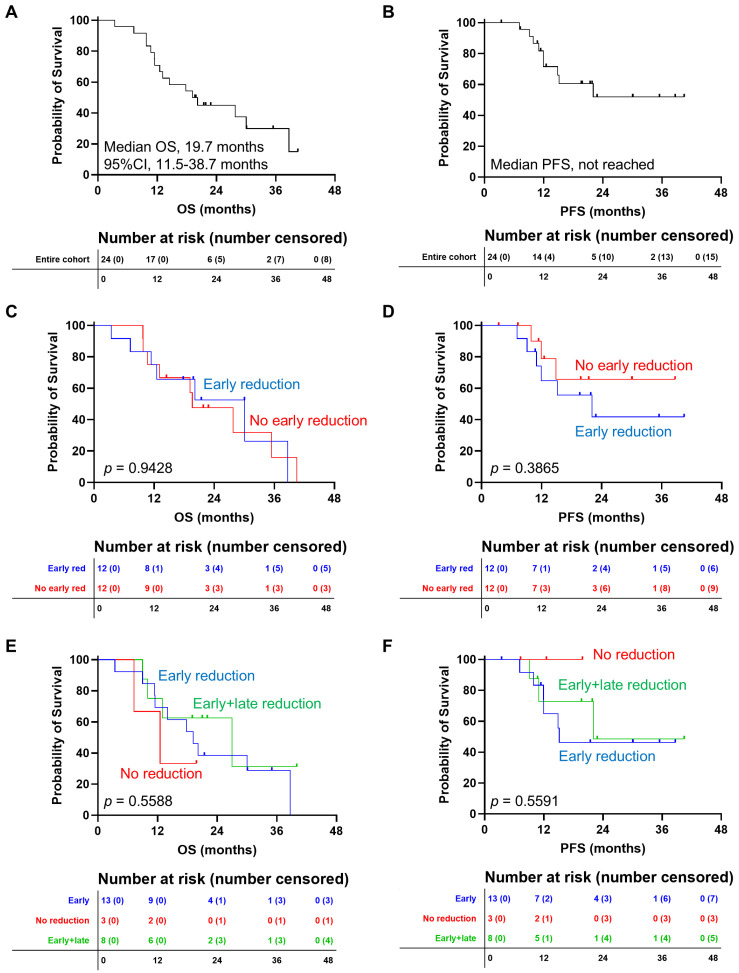
Clinical outcomes of unfit AML patients receiving azacytidine + venetoclax. (**A**) Overall survival (OS) and (**B**) progression-free survival (PFS) of the entire cohort. Patients were also stratified based on early venetoclax reduction and (**C**) OS and (**D**) PFS were compared between groups. Moreover, patients were divided according to early-only or early- and late-dose adjustments, and (**E**) OS and (**F**) PFS were compared. The number of censored subjects at risk is shown.

The overall response rate (ORR = CR + CRi) to azacytidine + venetoclax was 87.5%, and 16 patients achieved a response already at the first re-evaluation (66.7%; 4 CR and 12 CRi). For the entire cohort, median OS was 19.7 months (95% CI; 11.5–38.7), while the median PFS was not reached (2-year PFS, 51.9%) (Figure 1A,B). Next, patients were first divided based on venetoclax reduction at the first re-evaluation, and no statistically significant variations were observed in OS (median OS, 19.6 months [95% CI, 9.8–35.5] vs. 30.1 months [95% CI, 7.3-n.v.], no reduction vs. reduction; *p* = 0.9428) and PFS (median PFS, not reached vs. 22.1 months [95% CI, 9.1-n.v.], no reduction vs. reduction; *p* = 0.3865) between groups (Figure 1C,D). Subsequently, patients were also divided according to early and late reduction, and no statistically significant differences were observed between groups in OS (median OS, 16.8 months vs. 20.2 months vs. 27.8 months, early vs. late vs. early and late reduction; *p* = 0.7568) and PFS (median PFS, 13.5 months vs. not reached vs. 22.1 months, early vs. late vs. early and late reduction; *p* = 0.4536), although a numerical trend toward lower relapse rates was observed in subjects with late (33.3%) or early and late reduction (37.5%) compared to those who had dose adjustment only at the first re-evaluation (75%) (*p* = 0.3014) (Figure 1E,F).

### 3.3. Safety of Reduced Venetoclax Dose

In 12 out of 24 of our patients, venetoclax was reduced from 28 days to 21 days, and in nine of them (75%) a further reduction was performed because of grade III neutropenia (41.7% of cases), grade IV neutropenia (25%), or febrile neutropenia (8.3% of subjects) at a median azacytidine plus venetoclax cycle of 4 (range, 3–8) (Table 2). No differences in clinical characteristics were observed between patients who reduced venetoclax dose at the first re-evaluation and those who never reduced the dose or reduced it later during the clinical course; however, the small number of patients per group does not allow for definitive conclusions and adjustment for potential confounding factors (Table 3).

The incidence of infectious complications was low, and events were mostly recorded between the sixth and eighth cycle and were fever of unknown origin (*N* = 11), pneumonia (*N* = 2), or urinary tract infection (*N* = 1). Median time of infectious disease onset was at the eighth cycle in those subjects who did not reduce venetoclax at the first re-evaluation (95% CI, 1-n.v.) or at the sixth cycle in those who early reduced venetoclax (95% CI, 1–19), without statistically significant variations between groups (*p* = 0.9104).

Subsequently, patients were also divided according to early and late reduction, and patients without dose reduction (66.7%) or with early venetoclax dose adjustment (100%) tended to experience more frequently infectious complications compared to those with late (33.3%) or early and late reduction (44.4%) (*p* = 0.2173), although statistical significance was not reached because of the small sample size. In detail, the infectious disease rate was 33.3% in those subjects with early and late reduction and 58.3% in those with early-only adjustment, compared to 84% in the VIALE-A study. The mortality rate was 66.7%; however, infection-related mortality was very low (8.3%), (8.3%), with only one case of sepsis in a subject who had not reduced venetoclax dose at the first re-evaluation.

### 3.4. WT1-Based MRD Monitoring for Venetoclax Dose Adjustment

**Figure 2:**
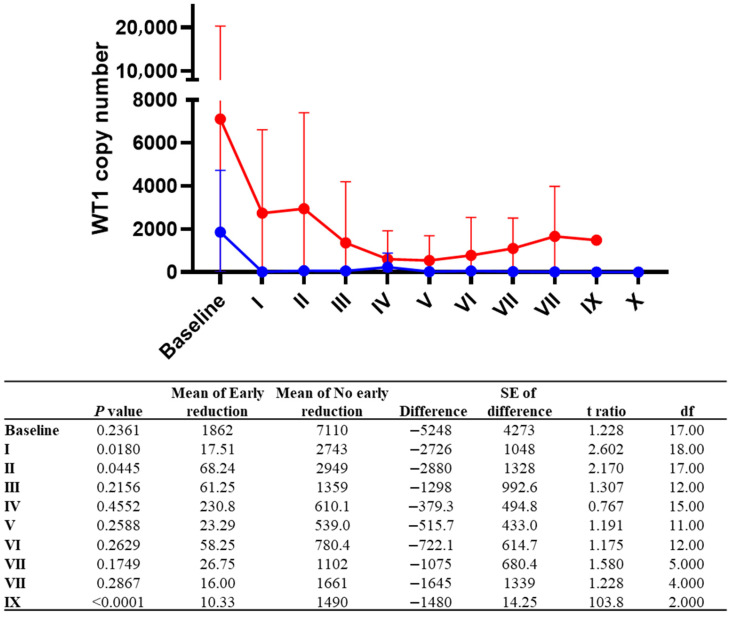
WT1 levels and monitoring. AML patients were divided based on early (blue line) or no early (red line) venetoclax reduction, and WT1 levels were compared between groups for each time point (roman numbers). *p*-values are exploratory (unadjusted for multiple comparisons).

In our cohort, circulating WT1 levels were employed as a surrogate biomarker of MRD monitoring, because of the lack of other well-known measurable markers and/or because of the patients’ frailties making a periodic BM evaluation challenging. Indeed, WT1-mRNA levels are used as a reliable, specific, and sensitive diagnostic and prognostic marker of AML and MDS, when specific molecular signatures are lacking (e.g., *NPM1* and *FLT3* wild type), as expression levels can mirror disease progression and identify MDS patients with shorter survival [33]. At diagnosis, only two subjects had low WT1 expression; however, patients with early venetoclax reduction tended to display lower WT1 levels at baseline (mean, 2212 vs. 7110 copies/10^4^ copies ABL; *p* = 0.1789), and throughout follow-up (Figure 2). Conversely, no statistically significant differences were observed in WT1 levels between patients with early and/or late reduction (all *p* > 0.05). However, the number of subjects in each group and timepoint is limited to clearly detect significant differences.

## 4. Discussion

AML is the most common acute leukemia in adults and older individuals and carries a poor prognosis, especially in those subjects unfit for intensive chemotherapy and subsequent allogeneic hematopoietic stem cell transplantation [37]. Elderly AML patients have significantly lower 5-year survival rates, ranging from ~25% in adults aged 60–65 to <10% in those 70 or older [38,39]. Therefore, patient fitness is a crucial clinical decision-making criterion for treatment strategy choice.

Venetoclax, a Bcl-2 inhibitor, has significantly changed clinical outcomes of these subjects, and in combination with HMAs is now considered the standard-of-care treatment for older AML patients or those who cannot undergo intensive chemotherapy [40]. Using this therapeutic regimen with venetoclax administered daily in 28-day cycles, 66.4% of older/unfit AML patients could reach a CR, with a median OS of 14.7 months, longer than that observed in the placebo plus azacytidine (CR rate, 28.3%, and median OS, 9.6 months) [11]. However, adverse events are frequent, including grade III–IV thrombocytopenia (45%), neutropenia (42%), and infections (85%), which negatively influence clinical outcomes [11,41]. Therefore, venetoclax-modified regimens are increasingly studied to reduce the incidence of toxicity without affecting clinical efficacy in an already frail AML population [18,42,43,44]. Results from a previous retrospective study have shown that CR/CRi rates are similar regardless of the venetoclax cycle duration (62–68%) in newly diagnosed AML patients treated with frontline venetoclax plus HMA, as well as relapse rates (33–41%) [11].

In our real-life retrospective observational case series study, unfit older AML patients were treated with azacytidine plus venetoclax, and MRD monitoring was carried out by flow cytometry immunophenotyping according to ELN guidelines [32], and by circulating WT1 expression measurement at every cycle. Based on hematological responses, MRD negativity, and neutropenia at the first re-evaluation, patients were switched to a reduced venetoclax dose (21 days) if they were in CR/CRi, with normal WT1 levels, and/or if they developed grade III or higher neutropenia. Half of the patients met these criteria for venetoclax reduction, and all of them were in CR/CRi at the time of the first re-evaluation; conversely, subjects who did not change the venetoclax schedule showed a lower CR/CRi rate (63%; *p* = 0.0373), with similar duration of response (median time, 11 months vs. 14 months, no reduction vs. early reduction; *p* = 0.5113). In other studies, CR/CRi rates are also similar regardless of ELN risk categories and cytogenetic alterations (69–81%) [11]. In our cohort, clinical and prognostic characteristics were balanced between groups, thus minimizing potential confounding effects. Indeed, AML patients with adverse risk showed similar OS regardless of venetoclax dose modifications (median OS, 11.5 months vs. 27.8 months, 28-day cycle vs. 21-day cycle; *p* = 0.2316). Our results support previous findings showing that venetoclax treatment duration does not influence response rates in patients harboring one or more unfavorable mutations; however, a subgroup analysis in *TP53*-mutated AML was not performed because only one subject was present in our cohort. Moreover, we support the evidence that a shortened venetoclax course may be as effective as the 28-day administration in inducing CR/CRi in unfit AML patients without compromising clinical outcomes [45]. In addition, the reported median OS is comparable between patients who receive venetoclax at various time courses (median OS, 18.6 months vs. 21.3 months vs. 13.2 months, 14 days vs. 21 days vs. 28 days; *p* = 0.94) [44]. Similarly, no differences in median OS between our patients without reduction or with early and/or late dose adjustment were observed, supporting the use of shortened venetoclax for treatment of unfit AML patients.

Shorter venetoclax regimens favor faster hematologic recovery, with improved platelet and neutrophil counts over time [46]. Infection-related complications require proactive management through early identification and preventive measures to reduce potential risks and mortality; therefore, continuous surveillance is essential to ensure patient safety and optimize therapeutic outcomes [45]. In our case series study, the infectious complication rate in early and late venetoclax dose adjustment was lower than that reported in the VIALE-A trial (33.3% vs. 84%, respectively) and tended to be lower than that observed in those AML patients with early or late adjustment. Common hematological toxicities, such as anemia, neutropenia, and thrombocytopenia, continue to pose substantial challenges in the management of unfit/frail AML, necessitating careful monitoring and supportive interventions, like transfusions [11]. In previous studies, incidence rates of grade III–IV neutropenia and thrombocytopenia are comparable regardless of venetoclax duration [18], although a tendency to a higher infection rate could be observed in patients receiving a 28-day course of venetoclax [43,44,45,46,47]. In our cohort, grade III neutropenia was documented in 41.7% of cases at the first re-evaluation and led to a venetoclax dose reduction in all cases; however, in eight of these subjects, four displayed persistent grade III neutropenia, three worsening to grade IV, and one febrile neutropenia, and all of them required further dose adjustment. On the other hand, neutropenia was documented in 58% of our AML subjects who did not adjust venetoclax at the first re-evaluation, thus continuing with the 28-day course until they developed grade III (five cases) or IV neutropenia (two subjects). Therefore, incidence rates of neutropenia were similar between AML patients treated with various venetoclax course duration (*p* = 0.1779). Cardiovascular events could also be documented during venetoclax for AML treatment, although limited literature is present [48]. In our series, two deaths occurred due to heart failure (8.3%), without a causality-certified relationship with venetoclax use. Because cardiovascular events are possible during venetoclax administration, strict surveillance is required for early identification and treatment.

Wilms tumor 1 (WT1) is expressed at low levels in normal progenitors, while it is overexpressed in normal hematopoietic stem cells, in the majority of AML, and at disease relapse after allogeneic stem cell transplantation [49,50,51], as BM blast count by flow cytometry is positively associated with WT1 levels in AML and MDS [33]. For these reasons, WT1 gene expression is often used as a surrogate biomarker of MRD in AML and MDS when another well-established molecular marker is not available [33]. In addition, WT1-mRNA levels are not correlated with the presence of cytogenetic abnormalities, while patients with low WT1 expression show a significantly higher mutational burden, with a median of 3.4 mutations per patient, compared to AML populations [25,33]. AML with *NPM1* or *FLT3* mutation and low WT1 expression have more clonal hematopoiesis- or MDS-related AML mutations; however, these subjects show an OS comparable to that expected in AML with myelodysplasia [25]. Moreover, WT1-based MRD positivity can independently predict response (Hazard Ratio, 7.4) and poor disease-free survival and OS after stem cell transplantation and is also associated with disease recurrence in patients with unfavorable risk and in those with normal karyotype without *NPM1* mutations before allogeneic stem cell transplantation [26,27]. From our preliminary data, WT1 levels tended to remain elevated in patients with disease persistence, while low WT1 levels were more frequently associated with sustained hematological response, thus supporting the potential role of this molecular marker in monitoring MRD in the PB when other well-known molecular alterations are present.

Our study has several limitations: (i) the small number of subjects due to the retrospective case series nature of our study (thus subgroup analysis was not possible, including comparisons between populations with different genomics features); (ii) the lack of a priori power analysis and of a minimum detectable effect calculation; (iii) the absence of a longer follow-up period to assess short- and long-term efficacy and safety; and (iv) the lack of blood venetoclax concentration monitoring, which could be inversely related to body surface area, thus greatly influencing the therapeutic dosage. Therefore, our preliminary findings warrant cautious interpretation and underscore the need for larger prospective studies to confirm these observations.

## 5. Conclusions

AML is the most common acute leukemia in adults and older individuals, often with a poor prognosis, especially in those unfit for intensive chemotherapy or stem cell transplantation, as 5-year survival significantly drops with aging. While intensive chemotherapy and HMAs outperform palliative care, outcomes remain poor for those ineligible for these approaches. Venetoclax combined with HMAs or low-dose cytarabine has become the standard of care for elderly or unfit AML patients, improving CR/CRi rates and median OS [9,10,11]. However, high rates of adverse events, including hematological toxicities and infectious complications, are common and negatively affect clinical outcomes. Reduced-dose venetoclax regimens are being explored to decrease toxicity without reducing efficacy [10,21,22]. Our preliminary results add evidence for the effective use of reduced-dose venetoclax (from 28 to 21 days per cycle) in unfit/frail AML patients without affecting response rates, survival, or incidence of side effects. In addition, WT1 expression could represent a reliable candidate marker for MRD monitoring when other molecular alterations are not present. However, larger prospective trials are needed for confirmation.

## Figures and Tables

**Table 1:** Patients’ characteristics at diagnosis.

Characteristics	*N* = 24
Median age, years (range)	74 (68–80)
M/F	17/7
Diagnosis, *n* (%)	
AML de novo	7 (29.2)
AML therapy-related	1 (4.1)
AML MDS-related	16 (66.7)
2022 ELN risk category, *n* (%)	
Favorable	4 (16.7)
Intermediate	5 (20.8)
Adverse	15 (62.5)
Median comorbidities, *n* (range)	2 (0–6)
Comorbidities, *n* (%)	
Solid tumors	3 (12.5)
Other hematological diseases	5 (20.8)
Hypertension	12 (50)
Dyslipidemia	6 (25)
Cardiovascular diseases	10 (41.7)
COPD	5 (20.8)
Hypothyroidism	2 (8.3)
Obesity	2 (8.3)
BPH	6 (25)
Diabetes	5 (20.8)
CKD	2 (8.3)
Others	8 (33.3)
Median WT1 levels, copy number (range)	
Peripheral blood	1388 (38–41,660)
Bone marrow	2815 (28–6501)
Cytogenetics abnormalities, *n* (%)	
Normal	8 (33.3)
del (5q)	2 (8.3)
Single	6 (25)
Complex karyotype	2 (8.3)
Failed culture	4 (16.7)
Not performed	2 (8.3)
Somatic mutations, *n* (%)	
*ASXL1*	7 (29.2)
*TET2*	6 (25)
*SRSF2*	5 (20.8)
*DNMT3A*	6 (25)
*IDH1/2*	7 (29.2)
*NPM1*	5 (20.8)
*RUNX1*	5 (20.8)
*TP53*	2 (8.3)
*KRAS*/*NRAS*	3 (12.5%)
Other genes	6 (25)
Previous therapies, *n* (%)	4 (16.7)
Cytarabine + gilteritinib	1 (4.2)
Liposomal Daunorubicin/Cytarabine	2 (8.3)
3 + 7 + gemtuzumab ozogamycin	1 (4.2)
Relapses, *n* (%)	9 (37.5)
Deaths, *n* (%)	16 (66.7)
PD	11 (45.8)
Cardiovascular events	2 (8.3)
Sepsis/ARDS	2 (8.3)
Secondary malignancy	1 (4.2)
Median time to first re-evaluation, days (range)	60 (31–410)
Best response, *n* (%)	
CR/CRi	14 (58.3)/7 (29.2)
PR	0
SD/PD	2 (8.3)/1 (4.2)
Response at the first re-evaluation, *n* (%)	
CR/CRi	4 (16.7)/12 (50)
PR	3 (12.5)
SD	4 (16.7)
Median flow cytometry MRD, % (range)	0.8 (<0.01–90)
Median WT1 copies at re-evaluation, copies (range)	14 (0–10,796)
Response at the second re-evaluation, *n* (%)	
CR/CRi	5 (20.8)/10 (41.7)
PR	2 (8.3)
SD	2 (8.3)

Abbreviations. AML, acute myeloid leukemia; MDS, myelodysplastic syndromes; ELN, European LeukemiaNet; COPD, chronic obstructive pulmonary disease; BPH, benign prostatic hyperplasia; CKD, chronic kidney disease; WT1, Wilms tumor 1; PD, progressive disease; ARDS, acute respiratory distress syndrome; CR, complete response; CRi, CR with incomplete blood count recovery; PR, partial response; SD, stable disease; MRD, minimal residual disease.

**Table 2:** Characteristics of venetoclax dose reduction.

Characteristics	*N* = 24
Initial dosage, *n* (%)	
70 mg	9 (37.5%)
100 mg	14 (58.4%)
400 mg	1 (4.2%)
Early reduction, *n* (%)	12 (50%)
Causes of reduction, *n* (%)	
Grade III neutropenia	10 (41.7%)
Pneumonia	1 (4.2%)
CR	1 (4.2%)
Early and late reduction, *n* (%)	9 (75%)/12
Causes of reduction, *n* (%)	
Grade III neutropenia	5 (41.7%)
Grade IV neutropenia	3 (25%)
Fever	1 (8.3%)
Late reduction, *n* (%)	8 (33.3%)
Causes of reduction, *n* (%)	
Grade III neutropenia	5 (62.5%)
Grade IV neutropenia	2 (25%)
CR	1(12.5%)
Median cycle of late reduction, *n* (range)	4 (3–8)

Abbreviations. CR, complete response.

**Table 3:** Clinical characteristics between groups.

Characteristics	Dose Unchanged at First Re-Evaluation *N* = 12	Early Dose Reduction *N* = 12	*p*-Value
M/F	8/4	9/3	>0.9999
Diagnosis, *n* (%)			>0.9999
AML de novo	3	4	
AML therapy-related	1	0	
AML MDS-related	8	8	
2022 ELN risk category, *n* (%)			0.7144
Favorable	3	1	
Intermediate	2	3	
Adverse	7	8	
Median comorbidities, *n* (range)	2 (1–6)	3 (0–5)	0.6416
Median WT1, copy number (range)			
Peripheral blood	3455 (38–41,660)	1204 (61–9504)	0.3119
Bone marrow	5088 (3675–6501)	294 (28–3961)	0.1731
Cytogenetics abnormalities, *n* (%)			0.5448
Normal	4	4	
Single	4	4	
Complex karyotype	2	0	
Failed/Not performed	2	4	
Previous therapies, *n* (%)	2	2	>0.9999
Relapses, *n* (%)	3	6	0.4003
Deaths, *n* (%)	9	7	0.6668
PD	6	5	
Others	3	2	
Early reduction, *n* (%)	9	8	

Abbreviations. AML, acute myeloid leukemia; MDS, myelodysplastic syndromes; ELN, European LeukemiaNet; WT1, Wilms tumor 1; PD, progressive disease.

## Data Availability

Data are available upon request to the authors.
